# Palliative care for in-patient malignant glioma patients in Germany

**DOI:** 10.1007/s11060-024-04611-8

**Published:** 2024-03-20

**Authors:** Larissa Fink, Birgitt van Oorschot, Christiane von Saß, Maxine Dibué, Marie-Therese Foster, Heidrun Golla, Ronald Goldbrunner, Christian Senft, Aaron Lawson McLean, Martin Hellmich, Nazife Dinc, Raymond Voltz, Heiner Melching, Christine Jungk, Marcel A. Kamp

**Affiliations:** 1 Center for Palliative and Neuro-palliative Care, Brandenburg Medical School Theodor Fontane, Faculty of Health Sciences Brandenburg, Am Seebad 82/83, 15562 Rüdersdorf bei Berlin, Germany; 2 Interdisciplinary Center for Palliative Medicine, University Hospital Würzburg, Würzburg, , Germany; 3 Department of Neurosurgery, Goethe University Hospital, Frankfurt am Main, Germany; 4 Department of Palliative Medicine, Faculty of Medicine and University Hospital, University of Cologne, Cologne, Germany; 5 Center of Neuro-Oncology, Department of Neurosurgery, Jena University Hospital, Friedrich-Schiller-University, Jena, Germany; 6 Center for Neurosurgery, Department of General Neurosurgery, University of Cologne, Cologne, Germany; 7 Institute of Medical Statistics and Computational Biology (IMSB), Faculty of Medicine and University Hospital Cologne, University of Cologne, Cologne, Germany; 8 Clinical Trials Centre Cologne (CTCC), Faculty of Medicine and University Hospital, University of Cologne, Cologne, Germany; 9 Center for Integrated Oncology Aachen Bonn Cologne Düsseldorf (CIO ABCD), Faculty of Medicine and University Hospital, University of Cologne, Cologne, Germany; 10 Center for Health Services Research (ZVFK), Faculty of Medicine and University Hospital, University of Cologne, Cologne, Germany; 11 German Association for Palliative Care Medicine, Berlin, Germany; 12 Department of Neurosurgery, University Hospital Heidelberg, Heidelberg, Germany; 13 Department of Neurosurgery, Medical Faculty, Heidelberg University, Heidelberg, Germany

**Keywords:** Palliative care, Neuropalliative care, Malignant glioma, Glioblastoma, Early integration, Predictive factors

## Abstract

**Objective:**

Malignant gliomas impose a significant symptomatic burden on patients and their families. Current guidelines recommend palliative care for patients with advanced tumors within eight weeks of diagnosis, emphasizing early integration for malignant glioma cases. However, the utilization rate of palliative care for these patients in Germany remains unquantified. This study investigates the proportion of malignant glioma patients who either died in a hospital or were transferred to hospice care from 2019 to 2022, and the prevalence of in-patient specialized palliative care interventions.

**Methods:**

In this cross-sectional, retrospective study, we analyzed data from the Institute for the Hospital Remuneration System (InEK GmbH, Siegburg, Germany), covering 2019 to 2022. We included patients with a primary or secondary diagnosis of C71 (malignant glioma) in our analysis. To refine our dataset, we identified cases with dual-coded primary and secondary diagnoses and excluded these to avoid duplication in our final tally. The data extraction process involved detailed scrutiny of hospital records to ascertain the frequency of hospital deaths, hospice transfers, and the provision of complex or specialized palliative care for patients with C71-coded diagnoses. Descriptive statistics and inferential analyses were employed to evaluate the trends and significance of the findings.

**Results:**

From 2019 to 2022, of the 101,192 hospital cases involving malignant glioma patients, 6,129 (6% of all cases) resulted in in-hospital mortality, while 2,798 (2.8%) led to hospice transfers. Among these, 10,592 cases (10.5% of total) involved the administration of complex or specialized palliative medical care. This provision rate remained unchanged throughout the COVID-19 pandemic. Notably, significantly lower frequencies of complex or specialized palliative care implementation were observed in patients below 65 years (*p* < 0.0001) and in male patients (p_adjusted_ = 0.016). In cases of in-hospital mortality due to malignant gliomas, 2,479 out of 6,129 cases (40.4%) received specialized palliative care.

**Conclusion:**

Despite the poor prognosis and complex symptomatology associated with malignant gliomas, only a small proportion of affected patients received advanced palliative care. Specifically, only about 10% of hospitalized patients with malignant gliomas, and approximately 40% of those who succumb to the disease in hospital settings, were afforded complex or specialized palliative care. This discrepancy underscores an urgent need to expand palliative care access for this patient demographic. Additionally, it highlights the importance of further research to identify and address the barriers preventing wider implementation of palliative care in this context.

## Introduction

Malignant gliomas are the most prevalent intracerebral malignant brain tumors [16]. Despite advancements in diagnostics and therapy, they remain incurable, offering a very restricted prognosis. Glioblastoma is regarded as one of the most malignant forms of cancer. The survival of patients with malignant gliomas hinges on several factors, including overall health status, age, tumor location, and genetic and epigenetic tumor traits [15]. When untreated, individuals with glioblastoma face a prognosis of just 3 months. However, with a multimodal treatment approach encompassing surgery, radiotherapy, and chemotherapy, the median survival often extends beyond 20 months [15, 26]. Certain patient subgroups, characterized by specific genetic traits and tailored treatments, have demonstrated median survivals of up to 48 months [7]. Anaplastic gliomas harboring IDH mutations exhibit varying prognostic outcomes according to their distinct molecular properties [29, 30]. While some individuals may experience a median survival of several years, it’s essential to recognize that these tumors are, in essence, incurable.

Malignant gliomas frequently carry a substantial symptom burden for both patients and their families. Following the comprehensive framework of palliative care, these symptoms encompass physical, psychological, social, and spiritual challenges [24]. On the physical front, patients may confront focal neurological deficits, headaches, signs of intracranial pressure, or seizures. Psychologically, they may grapple with fear, depression, and existential concerns, while on the social plane, they may experience restrictions in family life, reduced social engagement, and caregiving difficulties [10, 13, 22, 23]. The diagnosis of a malignant disease represents a profoundly distressing experience for patients and their families, leading to a significant deterioration in the patient’s quality of life, functional decline, and ultimately death.

Hence, current guidelines advocate for the early integration of (specialized) palliative care [18, 31]. Additionally, the latest ASCO guidelines recommends the inclusion of palliative care for all patients with advanced tumor disease within 8 weeks of diagnosis [5]. While the necessity for early palliative care is well-recognized, actual clinical practices may diverge significantly. A retrospective analysis by Pando et al. of the US National Cancer Database, spanning the period 2004 to 2017, examined 85,380 glioblastoma patients [19]. Of these, only 2,803 (3.3%) were recorded as having received palliative care. However, this study’s scope included both ‘palliative treatments’ and ‘palliative care,’ encompassing a range of interventions from surgical and radiation therapies to drug-based tumor treatments and pain management. This raises critical questions about the effectiveness of these tumor-specific ‘palliative’ treatments in symptom alleviation and whether they might precipitate new symptoms that require genuine palliative care interventions. Notably, less than 15% of these patients received pain management, highlighting its limited role in the broader spectrum of palliative care. Additionally, a similar proportion of patients received an unspecified form of palliative care, indicating a need for clearer categorization and understanding of palliative care practices in this patient population.

Palliative care is organized differently in different countries. In Germany, there are different forms of palliative care. In principle, a distinction can be made between outpatient and inpatient palliative care and between general and specialized palliative care. General palliative care is usually the responsibility of the primary care physicians. In the outpatient setting, these are usually general practitioners and general palliative care nurses, while in the inpatient setting it is provided by doctors with basic knowledge of palliative care and nurses. Specialized palliative care is provided to palliative care patients with a particularly complex symptom burden and complex care needs. This care is typically provided by dedicated multi-professional teams in both inpatient and outpatient settings. In the outpatient context, multi-professional Specialized Ambulance Palliative Care (SAPC, in German SAPV) teams - comprising at least doctors, nurses and coordinators - provide 24/7 symptom management support and complement existing services such as GPs, specialists or nursing services. SAPV provides palliative care at home, in nursing homes and often in hospices. Specialized inpatient palliative care is provided by multi-professional teams specializing in palliative care: Inpatient consultation services support general hospital wards in the symptom management of complex palliative care patients, who are often still receiving tumor-specific treatment. This is one way of integrating palliative care at an early stage. However, palliative care units focus primarily on symptom control rather than tumor-specific treatment. Meanwhile, more than 70% of patients transferred to a palliative care unit die there. In Germany, end-of-life care is further provided within inpatient hospices, where the exclusive approach revolves around symptom-oriented therapy, and anti-tumor treatments are excluded.

The proportion of glioblastoma patients receiving palliative care has not been established for Germany or other European countries. The collection of comprehensive data on the management of patients with malignant glioma in Germany poses a significant challenge. Nevertheless, for the inpatient domain, a valuable dataset remains largely untapped in the form of hospitals’ quality reports, particularly concerning glioma patients. This study seeks to address two key aspects: first, the number of patients with malignant glioma who died within a hospital setting or were relocated to a hospice facility between 2019 and 2022, and second, the count of these patients who have received specialized palliative care interventions.

## Methods

### Ethics approval and data availability

This study adhered strictly to the ethical principles delineated in the 1964 Helsinki Declaration and its subsequent amendments. It exclusively utilized publicly available data, thus obviating the need for individual patient consent. Consistent with this approach, and in alignment with prevailing local, state, and federal regulations, explicit approval from an institutional ethics board was not requisite for this research.

### Study design

In this cross-sectional study, we retrospectively assessed the data retrieved from the Institute for the Hospital Remuneration System (*Institut für das Entgeltsystem im Krankenhauswesen, InEK GmbH, Siegburg, Germany*) for the years 2019 to 2022.

### Setting and data source

A performance-oriented and flat-rate remuneration system was introduced for general hospital services in German hospitals under Section 17b of the Hospital Financing Act. This system, based on the German Diagnosis Related Groups (G-DRG) System, assigns a specific DRG flat rate for reimbursement to each inpatient treatment case. The responsibility for implementing and maintaining this system has been entrusted to InEK GmbH by key stakeholders in the healthcare sector, including the German Hospital Association, central health insurance associations, and the Association of Private Health Insurance.

German hospitals are required to furnish performance data to the InEK data centers. This obligation has been further intensified in response to the SARS-CoV-2 pandemic and the Second Civil Protection Act *(§ 21 Krankenhausentgeltgesetz - Hospital Remuneration Act*). InEK prepares and compiles the data, which is subsequently accessible through the InEK data browser [9]. The dataset is constrained to demographic particulars, primary and secondary diagnoses, and procedures. To safeguard data privacy, ages are grouped into specific age categories, while hospitals are categorized according to their respective bed capacity and sponsorship affiliation. Additionally, any analysis outcomes with a count lower than four are not presented, in adherence to data protection measures.

### Cohort/Participants

The InEK data pertains to hospital inpatient case data, but it cannot be equated with the corresponding count of patients. The population within the data delivery period excludes accompanying individuals or cases solely occurring outside the hospital. However, pre- and post-hospital services are encompassed within the datasets, unless they are subject to separate payment arrangements.

For the current analysis, we examined the following patient cohort: (1) hospital cases diagnosed with a malignant glioma, designated by the International Statistical Classification of Diseases and Related Health Problems (ICD)-10 code C71, and (2) patients aged 18 and above. We considered hospital cases for patients with a C71 diagnosis, whether it was their primary or secondary diagnosis. Furthermore, we extracted data separately for two comparable cohorts of patients: those who passed away in the hospital (discharge reason 09: death) and those who were directly transferred from the hospital to a hospice (discharge reason 11: transfer to a hospice).

### Variables and definitions

The following data were extracted from the InEK dataset for the aforementioned cohorts:

1. The total number of cases for each cohort, including cases with C71 codes as both the primary and secondary diagnosis.
2. Sex distribution.
3. Age distribution.
4. Distribution of treating hospitals, including hospital bed size and sponsorship.
5. The count of relevant secondary diagnoses.
6. The count of relevant procedures.

To calculate the number of cases for patients with a C71 diagnosis, we retrieved the counts of patients with C71 as either the primary or secondary diagnosis and those with double-coded both primary and secondary diagnoses. We then subtracted the number of double-coded cases from the sum of cases in which a C71 code appeared as either the primary or secondary diagnosis.

All procedures were identified and characterized using the Operation- and Procedure Code (Operationen- und Prozedurenschlüssel, OPS).

Using the OPS codes, three different types of specialized palliative care can be distinguished and refunded:

1. *OPS code 8-982: Complex palliative care treatment*. Essential components of this treatment encompassed specialist management by a practitioner with additional qualifications in palliative medicine, the implementation of a standardized palliative medicine basic assessment (PBA), comprehensive care for symptom control and psychosocial stabilization, the development and documentation of individualized treatment plans and progress records, weekly interdisciplinary team meetings, and the engagement of multi-professional teams.
2. *OPS code 8-98e: Specialized inpatient complex palliative care treatment*. For this code, in additional structural prerequisites are required: Evidence of an independent palliative care unit, the composition of the multi-professional specialist team with appropriate qualifications, the guarantee of a 24/7 medical on-call service for qualified symptom control, including the availability of specialized palliative care treatment procedures with equipment. The additional qualification in palliative care is required for both medical and nursing management, each with at least 6 months’ professional experience in specialist palliative care.
3. *OPS code 8-98 h: Specialized palliative care inpatient service*. Similar criteria as for the OPS code 8-98e are imposed for specialized palliative care complex treatment administered by a specialized palliative care inpatient service as a support service for complex palliative care patients to other general medical wards.
4. *OPS code 1-773 or 1-774*. OPS code 1-773 describes a multidimensional palliative medicine screening and minimal assessment and OPS code 1-774 a standardized palliative medicine basic assessment. The palliative medicine screening and basic assessment are usually part of the palliative medicine codes described above.

Biopsies were designated by the OPS codes 1-510 / 1-511, while neurosurgical tumor removal was identified by the OPS codes 5–015. The calculation of inpatient drug tumor treatment rates involved summing the OPS codes 8-541 to 8-547 and 6 − 002 to 6–007. In a similar fashion, the inpatient radiotherapy treatment rate was determined by summing the OPS codes 8-522 to 8-523.

### Study size

The study size was determined by examining inpatient palliative care for patients with malignant gliomas during the period from 2019 to 2022.

### Statistics

We obtained the data in the form of an Excel file (Microsoft Excel for Mac; Version 16.78, Microsoft Cooperation, Redmond, Washington, USA) from the InEK data browser for the previously defined patient cohorts. We selected the variables described earlier for subsequent analysis. For the statistical analyses and graphs, we used Graph Pad Prism 9 for macOS (Version 9.5.0, GraphPad Software, Inc., La Jolla, USA).

We analyzed the data using descriptive statistics. Specifically, we calculated the proportion (%) of patients who either passed away or were transferred to a hospice, as well as the proportion of patients who received treatment, with a specific focus on palliative care treatments. We used Pearson’s chi-square test to test for differences in distributions of categorical variables, specifically gender and dichotomized age [20, 21].

In this study, a two-sided significance level of α = 0.05 was chosen. We performed a total of six statistical evaluations. To guard against error inflation due to multiple comparisons, we applied the sequentially rejective Bonferroni-Holm procedure, i.e. ordered p-values are compared to increasing significance levels α/k with k = 6, 5,…, 1 [8].

## Results

### Patient cohort

**Fig. 1:**
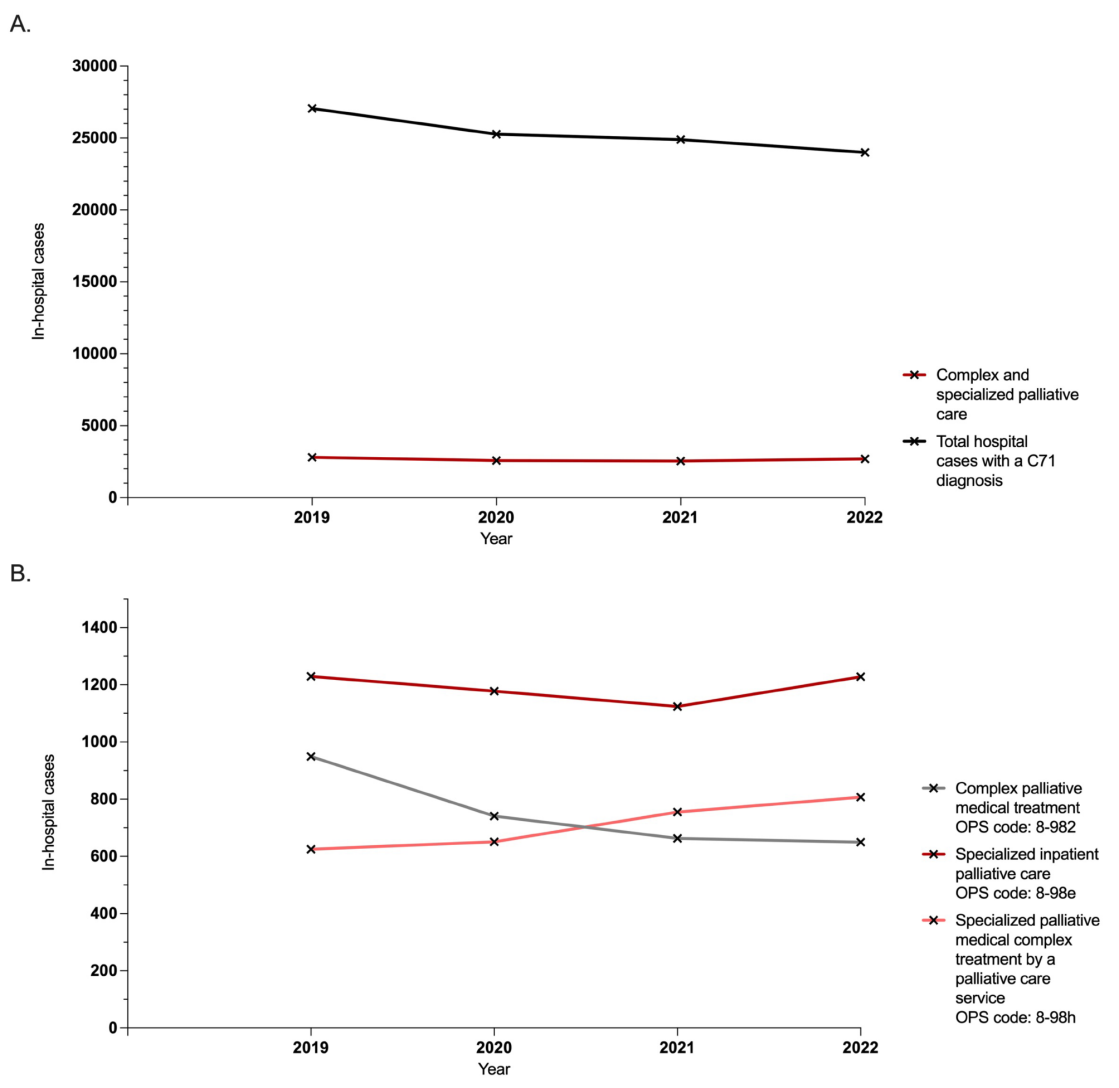
Total number of German hospital cases involving patients with malignant glioma from 2019 to 2022. **A**. illustrates the trend in inpatient cases involving malignant glioma patients in Germany from 2019 to 2022, along with the in-patients count receiving complex or specialized palliative care treatments. **B**. demonstrates the number of hospital cases receiving complex palliative medical treatment, specialized palliative medical care within a palliative care unit, and through consultation services

Between 2019 and 2022, German healthcare facilities treated a total of 101,192 hospital cases involving adult patients with malignant gliomas. Among these cases, healthcare professionals diagnosed a C71 ICD code as the primary diagnosis 78,898 times, as a secondary diagnosis 44,139 times, and as both a main and secondary diagnosis 21,845 times. The total number of hospital cases consistently decreased by 11% during the period under examination, going from 27,048 cases in 2019 to 23,996 cases in 2022 (Fig. 1).

**Fig. 2:**
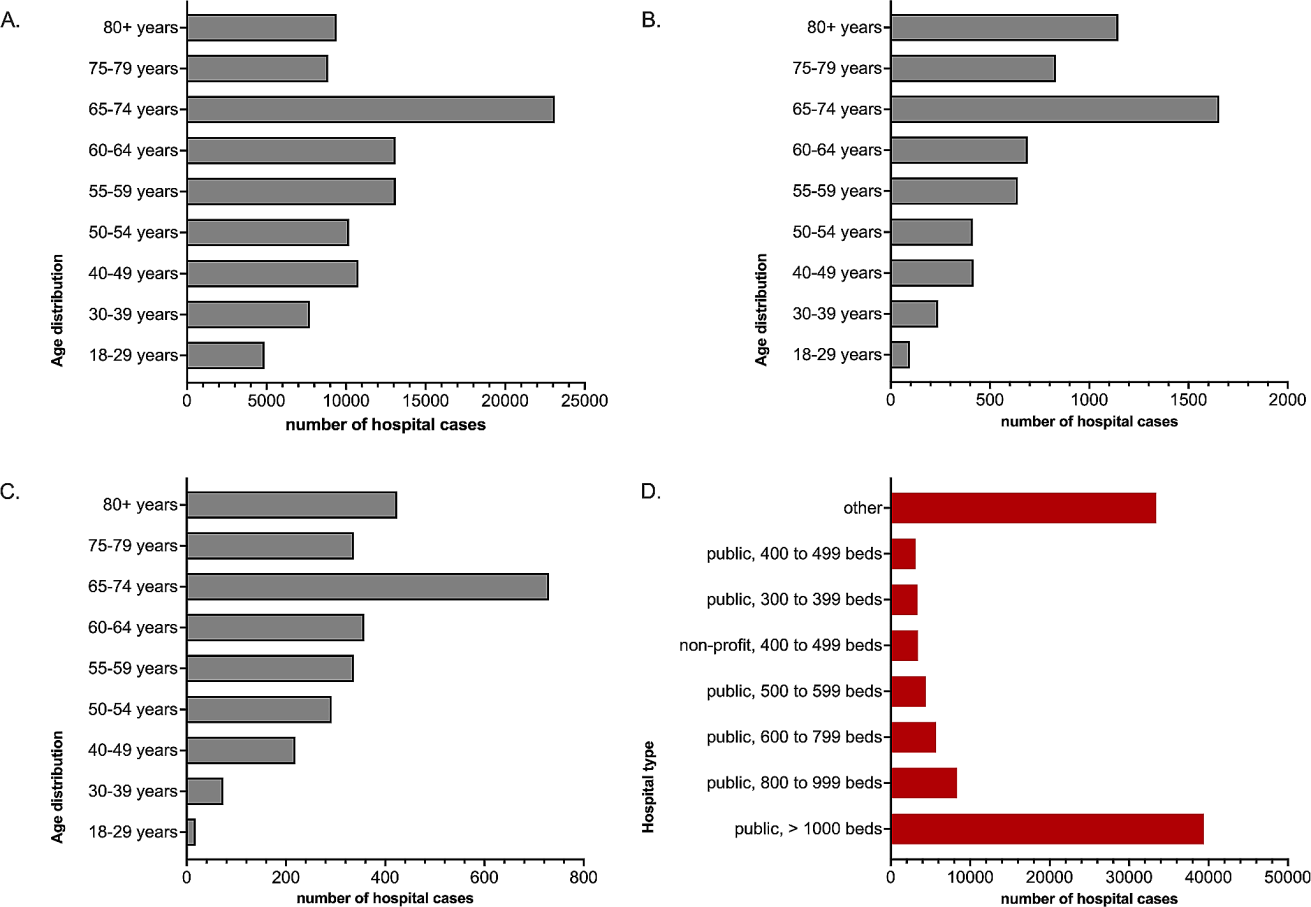
Age distribution and treating hospitals von patients suffering from malignant glioma. The figure shows the age distribution of the included malignant glioma patient population for the entire population (**A**.), the deceased patients (**B**.) and the patients discharged to a hospice (**C**.). Additionally, Figure **D**. gives an overview of the size and sponsorship of the treating hospitals

Overall, females represented 42.2% of hospital cases, with male patients comprising 57.8%. Among all hospital cases, 41,388 (40.9%) featured patients aged 65 years or older (Fig. 2). Table 1 displays the age and gender distribution as well as other demographic information. The majority of cases received treatment in public hospitals with over 1000 beds (39,424 hospital cases, 39%; Fig. 2), followed by public hospitals with a bed capacity between 800 and 999 (8,313 hospital cases, 8.2%).

**Table 1:** Overview about malignant glioma and palliative care treatment

	All malignant glioma patients						Malignant glioma patients died in hospital					Malignant glioma patients transition to hospice care				
	ICD or OPS code	2019	2020	2021	2022	**Total**	2019	2020	2021	2022	**Total**	2019	2020	2021	2022	**Total**
**Entire cohort**																
**All**	C71	27048	25262	24886	23996	**101192**	1643	1463	1445	1578	**6129**	702	670	663	754	**2789**
**Sex**							1643	1463	1445	1578						
Male		15585	14649	14281	13943	**58458**	1036	885	871	986	**3778**	393	363	360	415	**1531**
Female		11462	10613	10605	10053	**42733**	607	578	574	592	**2351**	309	307	303	339	**1258**
**Age**																
18–29 years		1425	1227	1199	1024	**4875**	32	21	17	27	**97**	5	4	2	7	**18**
30–39 years		2066	1825	1934	1908	**7733**	72	38	51	78	**239**	23	15	14	22	**74**
40–49 years		2971	2757	2626	2418	**10772**	107	97	100	113	**417**	59	50	60	50	**219**
50–54 years		2750	2717	2458	2263	**10188**	108	106	92	107	**413**	85	79	61	67	**292**
55–59 years		3407	3355	3338	3021	**13121**	148	165	167	160	**640**	97	85	87	68	**337**
60–64 years		3391	3119	3251	3354	**13115**	197	150	171	172	**690**	88	78	74	118	**358**
65–74 years		6060	5628	5674	5749	**23111**	442	396	399	418	**1655**	166	162	188	214	**730**
75–79 years		2710	2275	2032	1857	**8874**	266	220	176	170	**832**	96	90	72	79	**337**
80 + years		2268	2359	2374	2402	**9403**	271	270	272	333	**1146**	83	107	105	129	**424**
**Length of hospital stay**																
Short stay		2820	2358	2546	2354	**10078**	186	179	167	178	**710**	3	10	6	6	**25**
Intermediate stay		20822	19922	19623	18202	**78569**	1056	981	960	966	**3963**	393	368	384	374	**1519**
Long stay		3406	2982	2717	3440	**12545**	401	303	318	434	**1456**	306	292	273	374	**1245**
**Complex /specialized palliative care**																
No complex or specialized palliative care		24245	22692	22344	21311	**90592**	961	881	873	935	**3650**	311	265	263	322	**1161**
**All**		2803	2570	2542	2685	**10600**	682	582	572	643	**2479**	391	405	400	432	**1628**
Complex palliative medical care	8-982	949	741	663	650	**3,003**	182	161	123	139	**605**	130	119	97	106	**452**
Inpatient palliative care: specialized treatment	8-98e	1229	1,178	1,124	1,228	**4,759**	442	354	380	402	**1578**	230	244	272	272	**1018**
Specialized inpatient palliative care services	8-98h	625	651	755	807	**2,838**	58	67	69	102	**296**	31	42	31	54	**158**
**Sex**																
*Complex /specialized palliative care*		2803	2570	2542	2685	**10600**	682	582	572	643	**2479**	391	405	400	432	**1628**
Male		1553	1501	1413	1528	**5995**	424	348	324	397	**1493**	211	235	210	225	**641**
Female		1250	1069	1129	1157	**4605**	258	234	248	246	**986**	180	170	190	207	**747**
*Specialized palliative care*		1854	1829	1879	2035	**7597**	500	421	449	504	**1874**	261	286	303	326	**1176**
Male		1020	1068	1051	1165	**4304**	307	246	252	314	**1119**	149	162	161	169	**641**
Female		834	761	828	870	**3293**	193	175	197	190	**755**	112	124	142	157	**535**
**Age**																
*Complex /specialized palliative care*																
18–29 years		73	38	41	49	**201**	26	10	7	12	**55**	4	4	1	4	**13**
30–39 years		145	100	115	163	**523**	37	16	25	43	**121**	13	11	11	15	**50**
40–49 years		257	246	219	206	**928**	52	44	42	44	**182**	36	32	46	27	**141**
50–54 years		312	275	231	230	**1048**	56	49	37	48	**190**	54	55	46	45	**200**
55–59 years		295	334	316	330	**1275**	51	62	68	67	**248**	56	52	54	39	**201**
60–64 years		338	290	333	353	**1314**	84	63	66	69	**282**	50	39	38	76	**203**
65–74 years		718	636	654	721	**2729**	181	162	159	162	**664**	92	100	116	119	**427**
75–79 years		352	328	294	249	**1223**	95	89	71	66	**321**	50	56	40	43	**189**
80 + years		313	323	339	384	**1359**	100	87	97	132	**416**	36	56	48	64	**204**
*Specialized palliative care*																
18–29 years		55	30	30	43	**158**	20	7	7	10	**44**	2	4	0	2	**8**
30–39 years		97	76	95	124	**392**	27	15	20	34	**96**	8	6	10	12	**36**
40–49 years		178	178	157	163	**676**	36	31	33	29	**129**	27	27	35	19	**108**
50–54 years		222	199	177	176	**774**	45	37	28	35	**145**	38	43	36	33	**150**
55–59 years		201	237	232	242	**912**	40	49	52	48	**189**	37	35	40	28	**140**
60–64 years		230	211	245	273	**959**	59	39	50	59	**207**	39	30	30	59	**158**
65–74 years		466	450	460	551	**1927**	139	112	122	131	**504**	58	64	80	91	**293**
75–79 years		212	225	229	170	**836**	65	65	60	54	**244**	31	38	33	28	**130**
80 + years		193	223	254	293	**963**	69	66	77	104	**316**	21	39	39	54	**153**
**Complex palliative care**	**8-982**															
Total		949	741	663	650	**3003**	182	161	123	139	**605**	130	119	97	106	**452**
**Sex**																
Male		533	433	362	363	**1691**	117	102	72	83	**374**	62	73	49	56	
Female		416	308	301	287	**1312**	65	59	51	56	**231**	68	46	48	50	**212**
**Age**																
18–29 years		18	8	11	6	**43**	6	3	0	2	**11**	2	0	1	2	**5**
30–39 years		48	24	20	39	**131**	10	1	5	9	**25**	5	5	1	3	**14**
40–49 years		79	68	62	43	**252**	16	13	9	15	**53**	9	5	11	8	**33**
50–54 years		90	76	54	54	**274**	11	12	9	13	**45**	16	12	10	12	**50**
55–59 years		94	97	84	88	**363**	11	13	16	19	**59**	19	17	14	11	**61**
60–64 years		108	79	88	80	**355**	25	24	16	10	**75**	11	9	8	17	**45**
65–74 years		252	186	194	170	**802**	42	50	37	31	**160**	34	36	36	28	**134**
75–79 years		140	103	65	79	**387**	30	24	11	12	**77**	19	18	7	15	**59**
80 + years		120	100	85	91	**396**	31	21	20	28	**100**	15	17	9	10	**51**
**Specialized palliative care**																
*Inpatient palliative care: specialized treatment*	**8-98e**															
Total		1229	1,178	1,124	1,228	**4759**	442	354	380	402	**1578**	230	244	272	272	**1018**
**Sex**																
Male		692	699	633	708	**2732**	271	215	216	247	**949**	132	136	147	142	**557**
Female		537	479	491	520	**2027**	171	139	164	155	**629**	98	108	125	130	**461**
**Age**																
18–29 years		29	13	13	22	**77**	15	3	5	8	**31**	2	2	0	2	**6**
30–39 years		53	45	47	72	**217**	24	11	15	32	**82**	7	4	9	10	**30**
40–49 years		122	107	97	90	**416**	32	25	30	23	**110**	24	25	30	15	**94**
50–54 years		155	138	111	110	**514**	41	33	24	28	**126**	35	39	35	25	**134**
55–59 years		144	144	141	135	**564**	36	41	41	37	**155**	34	30	35	21	**120**
60–64 years		149	142	141	154	**586**	53	36	44	42	**175**	34	28	27	47	**136**
65–74 years		315	299	284	346	**1244**	124	96	106	104	**430**	54	53	70	84	**261**
75–79 years		135	150	130	109	**524**	58	54	51	45	**208**	23	31	29	24	**107**
80 + years		127	140	160	190	**617**	59	55	64	83	**261**	17	32	37	44	**130**
*Specialized inpatient palliative care services*	**8-98h**															
**All cases**		625	651	755	807	**2838**	58	67	69	102	**296**	31	42	31	54	**158**
**Sex**																
Male		328	369	418	457	**1572**	36	31	36	67	**170**	17	26	14	27	**84**
Female		297	282	337	350	**1266**	22	36	33	35	**126**	14	16	17	27	**74**
**Age** **0**																
18–29 years		26	17	17	21	**81**	5	4	2	2	**13**	0	2	0	0	**2**
30–39 years		44	31	48	52	**175**	3	4	5	2	**14**	1	2	1	2	**6**
40–49 years		56	71	60	73	**260**	4	6	3	6	**19**	3	2	5	4	**14**
50–54 years		67	61	66	66	**260**	4	4	4	7	**19**	3	4	1	8	**16**
55–59 years		57	93	91	107	**348**	4	8	11	11	**34**	3	5	5	7	**20**
60–64 years		81	69	104	119	**373**	6	3	6	17	**32**	5	2	3	12	**22**
65–74 years		151	151	176	205	**683**	15	16	16	27	**74**	4	11	10	7	**32**
75–79 years		77	75	99	61	**312**	7	11	9	9	**36**	8	7	4	4	**23**
80 + years		66	83	94	103	**346**	10	11	13	21	**55**	4	7	2	10	**23**

### Deceased malignant glioma patients within the hospital

Between 2019 and 2022, a total of 63,389,624 inpatient cases were treated in Germany (excluding psychiatric cases). Among these, 1,743,533 patients passed away, resulting in a notably lower mortality rate of 2.8%. In the same timeframe, 6,129 patients diagnosed with malignant gliomas passed away during their hospital stay. This corresponds to an average rate of 6.2% concerning the total number of hospital cases involving patients with malignant gliomas. The annual count of hospital mortalities for malignant glioma patients fluctuated from 1,445 to 1,643 patients (in 2021 and 2019, respectively), constituting between 5.8% (in 2021) and 6.6% (in 2022) of hospital cases for malignant glioma patients. Among these hospital cases, 1,233 patients underwent craniotomies (20.1% of 6,129), 746 patients had tumor resections (12.8%), 164 patients received inpatient chemotherapy or immunotherapy (2.7%), and 413 patients received a radiotherapy (6.7%).

During the same period, patients with malignant gliomas were transferred from the hospital to hospice in 2,789 cases, accounting for 2.8% of all hospital cases. The annual numbers remained relatively steady, ranging from 663 patients in 2021 to 754 patients in 2022.

### Specialized palliative care

In 2,950 hospital cases, representing 2.9% of all cases, either a multidimensional palliative care screening and minimal assessment or a standardized palliative care basic assessment (OPS code 1-773 or 1-774) was documented.

**Fig. 3:**
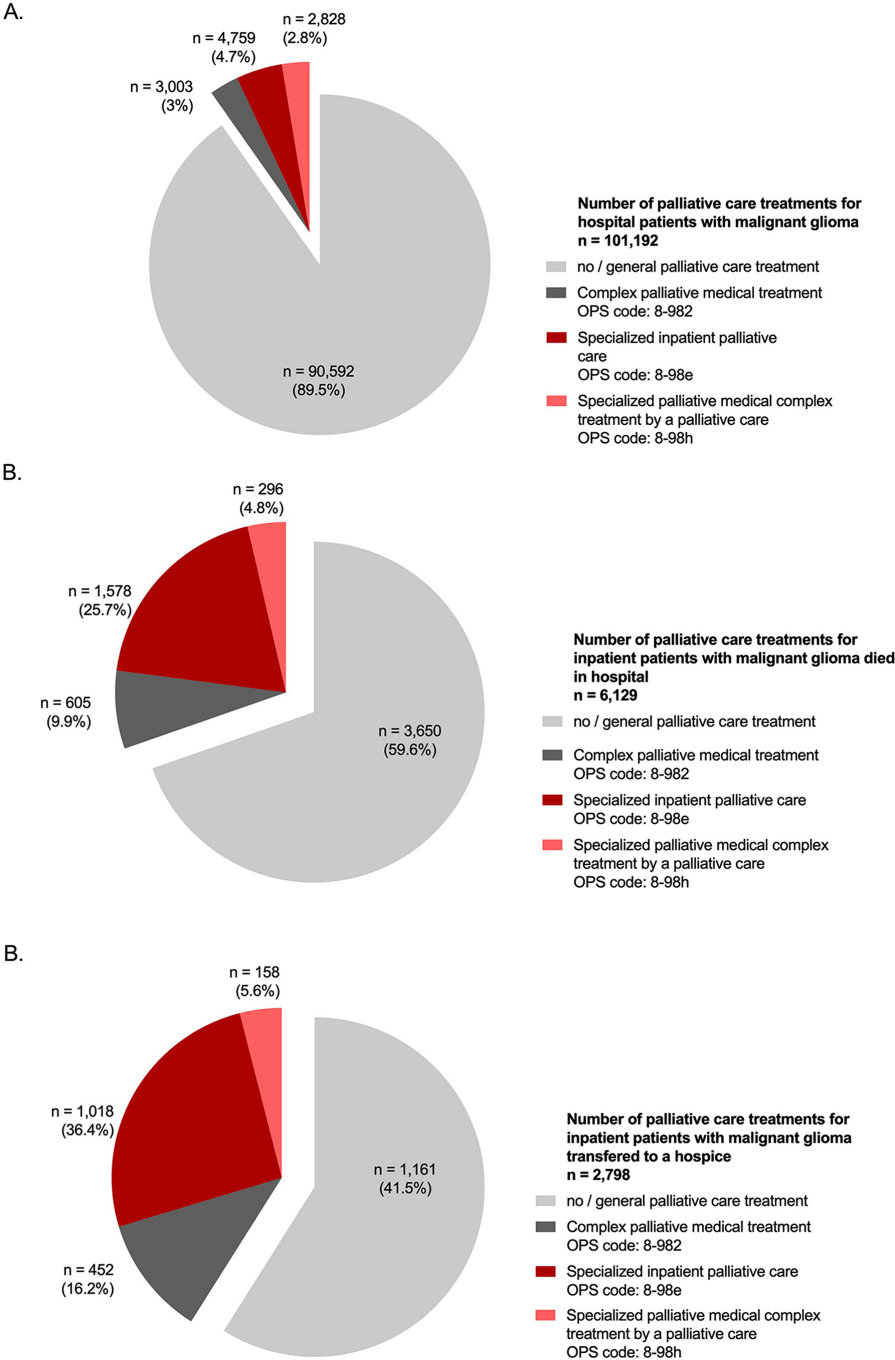
Complex and specialized palliative care in German malignant glioma patients. The figure illustrates the percentage of patients who underwent complex, specialized palliative care treatment either in a dedicated palliative care unit or via a consultation service. Figure A represents the data for the entire patient cohort, Figure B displays statistics for deceased patients, and Figure C presents information for patients discharged to hospice care

Specialized palliative care (codes 8-982, 8-98e, 8-98 h) was administered in 10,600 cases, representing 10.5% of all cases. The proportion of patients receiving specialized palliative care remained relatively stable throughout the study period, ranging from a minimum of 10.2% in 2020 to a maximum of 11.2% in 2022 (Fig. 3).

Complex palliative care treatment, as indicated by the 8-982 OPS code, was documented in 3,003 cases, accounting for 28.4% of all palliative care codes. Specialized palliative care for patients with malignant gliomas was provided in 7,597 cases, with 4,759 cases (44.9% of all palliative care codes) receiving specialized inpatient palliative care on a palliative care unit (OPS codes: 8-98e) at least those invoiced according to DRG, and 2,828 cases (26.7%) receiving specialized palliative care inpatient service by a multi-professional palliative care service (OPS codes 8-98 h). Palliative care utilization according to the ICD-10 Z51.5 code was recorded in 6,077 cases, representing 6% of all cases.

Out of the 6,129 patients diagnosed with malignant gliomas who passed away in the hospital, 605 individuals (9.9%) had prior complex palliative care treatment, while 1,578 patients (25.7%) received inpatient specialized palliative care treatment on a palliative care ward. Additionally, 296 patients received specialized palliative care through an internal consultation service (4.8%). Thus, complex or specialized palliative care was administered in 2,479 / 6,129 hospital cases in which patients with malignant gliomas passed away, making up 40.4% of the total. A palliative care assessment was documented in 541 cases, representing 8.8% of the total cases.

Among these hospital cases, 1,233 patients underwent craniotomy (constituting 20.1% of the 6,129 cases), 746 patients tumor resections (12.8%), 164 patients received inpatient chemotherapy or immunotherapy (2.7%), and 413 patients radiation therapy (6.7%) during the hospital stay in which they passed away.

### Sex and age-specific differences in care

Out of the total 101,192 inpatient cases encompassing patients diagnosed with malignant gliomas between 2019 and 2022, 42,733 cases (42.2%) pertained to female patients, and 58,458 cases (57.8%) involved male patients. Among these cases, 2,351 females and 3,778 males passed away (constituting 38.4% and 61.6%, respectively). In comparison to the overall count of cases concerning patients with malignant gliomas, there was a significantly higher number of instances in which male patients with gliomas experienced mortality (*p* < 0.0001). Furthermore, based on the total number of cases, there was a significant lower frequency of complex or specialized palliative care provided in hospital cases involving men (*p* = 0.016; p_adjusted_ = 0.016). This sex-specific difference in treatment was not significant for patients who died in hospital (*p* > 0.05).

As anticipated, the percentage of hospital cases resulting in patient mortality was notably higher in individuals aged ≥ 65 years (8.1% compared to 4%; *p* < 0.0001). Notably, complex or specialized palliative care was considerably more frequently initiated in elderly patients: Patients aged 65 years and older constitute 40.9% of the entire patient population. Among half of all hospital cases where patients diagnosed with malignant gliomas received complex or specialized palliative care, the patients were aged 65 years or older (*p* < 0.0001). Conversely, hospital cases with patients under 65 years of age were notably less likely to receive complex or specialized palliative care. Moreover, there was a trend towards a more frequent complex or specialized palliative care treatment in instances where patients passed away (30.2% compared to 27.8%; *p* = 0.019).

## Discussion

In this study, we present for the first time data on the inpatient specialized care of patients with malignant gliomas in Germany. The key findings from our current study are as follows:

1. Among the total of 101,192 hospital cases recorded between 2019 and 2022, which involved patients diagnosed with malignant gliomas, 6,129 patients (6% of all hospital cases with malignant glioma) passed away in the hospital, and an additional 2,798 cases (2.8%) saw patients being transferred to a hospice.
2. In 10,592 hospital cases, constituting 10.5% of the total, patients diagnosed with malignant gliomas received complex or specialized palliative medical care. This rate remained consistent even during the COVID-19 pandemic. Notably, there was a significant lower frequency of complex or specialized palliative care treatments observed in patients aged under 65 years and among male patients diagnosed with malignant gliomas.
3. Among patients who had malignant gliomas and passed away in hospital, specialized palliative care was provided in 2,479 out of 6,129 hospital cases, constituting 40.4%.

Malignant gliomas, especially IDH wild-type glioblastomas WHO grade 4, have a poor prognosis, often resulting in death within a few years. Prompt palliative care is recommended for these advanced tumors [5]. The persistent inquiry pertains to the coherence between the prescribed standard and the factual implementation of specialized palliative care. Our analysis reveals that specialized palliative care in Germany was provided to only 6% of hospital cases involving malignant gliomas, and approximately 40% of all hospital cases where patients with malignant gliomas passed away receiving palliative care. Similar findings exist in previous studies from other healthcare systems.

In the US National Cancer Database analysis (2004–2017), 2,803 out of 85,380 glioblastoma patients (3.3%) received so-called “palliative tumor treatment,” different from specialized palliative care provided by specialized professionals or multi-professional teams [19]. Another US study (1997–2016) using SEER-Medicare data found 15.24% of 10,812 GBM patients received palliative care, with an increasing trend from 3% (1997) to 43% (2015). The study identified palliative care by the ICD-9 code V66.7 or the ICD-10 code Z51.5 from Medicare data [33]. A separate US study on factors linked to hospice admission for malignant glioma patients revealed that 63% of 12,437 patients were enrolled in hospice before death, with certain demographics predicting enrollment [6]. Multivariable regression analysis revealed that advanced age, female gender, higher educational attainment, Caucasian ethnicity, and lower median household income were predictive of hospice enrollment [6]. In a retrospective cohort study in Victoria, Australia (2003–2009), 482 individuals with malignant glioma were analyzed. Palliative care, defined as hospital-based services, was utilized by 78% of 218 patients who passed [27]. Notably, 62% of these patients were admitted to a palliative care facility during their diagnostic admission [3, 27].

A retrospective observational study analyzed data from the Stockholm Region’s central data warehouse (VAL) from 2015 to 2019 [14]. Specialized palliative care, primarily delivered through advanced home care and hospital units, is highly regarded for its round-the-clock service by a multidisciplinary team. The study identified 780 patients with malignant brain tumors, showing an increasing trend in specialized palliative care utilization in the final year of life, peaking at 77%. However, no overall median or mean rate for specialized palliative care usage was provided for the entire cohort [14]. A recent Italian study investigating the treatment modalities for malignant glioma patients in the last three months did not evaluate palliative care [17]. A systematic review of 16 studies on glioblastoma patient service utilization revealed varying rates of advanced care documentation from 4 to 55% of cases, palliative care referral (about 40% of cases, and hospice initiation in 66–76% of patients [32].

The necessity of involving specialized palliative care varies depending on the severity of symptoms. For instance, depending on the study, symptoms like headaches, seizures, and fatigue can be observed in up to 90% of all patients, while personality changes, for example, occur in up to 60% of patients [18]. Consequently, specialized palliative care is not necessary for all malignant patients, but the observed rate of 10% in-patient complex / specialized palliative care in Germany between 2019 and 2022 appears comparatively low. As part of the certification of neuro-oncology, the German Cancer Society (*Deutsche Krebsgesellschaft, DKG*) now mandates the routine documentation of palliative care needs using recommended patient-reported outcome measures (PROMs) for patients with neuro-oncological conditions. The documentation of palliative care-related complaints and the ratio of patients receiving specialized palliative care to those dying from a tumor serve as quality indicators for effective, guideline-based treatment [11, 12]. In Germany, 40% of patients with malignant gliomas who passed away in hospitals received specialized palliative care. Additionally, Earle and colleagues established quality indicators for oncological treatment, specifying that at least 55% of cancer patients should access hospice services before their passing, with fewer than 8% being admitted within just 3 days of death [4].

Our study investigated specialized palliative care for malignant glioma patients, finding higher mortality rates among those aged ≥ 65, despite age often being considered in treatment guidelines. Older patients were more likely to receive specialized palliative care, consistent with trends in other studies, although patterns in palliative care for younger patients remain unclear. Further evaluation is needed to understand why younger patients receive fewer palliative care interventions during hospital stays in Germany.

Our study revealed significantly higher mortality rates among older glioma patients aged ≥ 65 years. This finding is not unexpected, as age is a well-established negative predictor of the overall survival in patients with malignant gliomas [15, 31]. Treatment protocols often consider a patient’s biological age in guidelines [31]. However, retrospective analyses have occasionally suggested more aggressive treatment approaches for older patients [1, 2]. Furthermore, we identified notable disparities in access to specialized palliative care: In the present and a previous study, older patients were significantly more likely to receive complex or specialized palliative care treatment compared to their younger counterparts [14]. Even though in-hospital mortality is markedly elevated among older patients, a thorough evaluation is essential to understand why younger patients receive significantly fewer palliative care interventions during their hospital stays in Germany. It’s doubtful that the symptom burden is substantially lower in younger malignant glioma patients. In everyday clinical practice, one notable aspect is that younger patients are often more inclined to undergo tumor-specific therapy solely based on their age, even when it may not be deemed useful or indicated.

Surprisingly, we noted a significant higher in-hospital mortality among male patients compared to the overall cases of individuals with malignant gliomas and a trend towards a substantially lower frequency of complex or specialized palliative care administered in hospital cases involving men. The underlying causes for these distinctions are currently unknown. While sex is not a definitive predictor of survival among malignant glioma patients [31], several studies suggest a survival advantage for women. An analysis of glioblastoma patients in the United States showed significant differences in survival based on gender, with women exhibiting superior overall survival, despite minor but significant differences in treatment and treatment timings [25]. Similarly, a study using the SEER database found lower cancer-specific survival rates for males compared to females across various age groups and Glioblastoma cancer stages [28]. In Sweden, no disparity in specialized palliative care delivery was observed, but data from the Swedish National Quality Registry for Primary Brain Tumors suggested a survival advantage for women, although deemed not clinically significant [14]. A US study identified a potential explanation for the better prognosis of women with malignant gliomas compared to men, discovering that standard therapy is more effective in female GBM patients and highlighting sex-specific molecular subtypes in GBM, suggesting personalized treatments based on sex-related molecular mechanisms to improve outcomes [34]. While acknowledging the molecular distinctions, findings from Stabellini’s research in the USA and our observations from Germany indicate that, apart from age-related variations, there might be gender-related dissimilarities in the management of oncological and palliative care for malignant gliomas. These disparities necessitate further investigation. Additionally, there should be a general discussion and research to ascertain whether inadvertent treatment differences exist based on age or gender.

However, differences in the treatment of individuals perhaps must exist: besides gender variances within the tumors, patients with malignant gliomas may also have gender-specific needs. Similarly, age-related necessities should be considered. Elderly individuals have distinct geriatric limitations and needs, yet these aspects are seldom captured in neuro-oncology and incorporated into decision-making processes. It is crucial to consider these unique patient needs, as well as their goals and wishes, during the decision-making process. This aspect is also a facet of personalized medicine.

### Limitations

We acknowledge several limitations in our current study: (1) In our analysis, we examined InEK data concerning hospital cases treated from 2019 to 2022. It is important to note that the count of hospital cases does not directly correspond to the actual number of patients. Deceased patients represent a unique case in this regard, as individuals generally experience only one instance of death, aligning the number of hospital cases with the number of patients in this specific scenario. (2) We calculated the case numbers by the difference between the sum of cases with either a primary or secondary diagnosis of an ICD-10 C71 code and the cases coded as both primary and secondary diagnoses. It is worth noting that the possibility exists that the secondary diagnosis C71 might have been coded multiple times within the same case. Consequently, the actual count of cases may be marginally lower. Nevertheless, we believe that the prevalence of such cases is likely to be minimal. (3) In Germany, not all palliative care units in operation bill for services via the DRG system. Approximately 70 palliative care units are classified as “special facilities” (*besondere Einrichtungen*), requiring a minimum of 5 beds with an average of 8 beds. An 8-bed unit typically handles around 250 in-patient cases annually, totaling approximately 17,500 cases across all palliative care entities. According to INEK data for 2021, 106,638 cases were billed as complex or specialized palliative care treatment (codes 8-982, 8-98e, 8-98 h), with 52,055 cases specifically designated as specialized palliative care treatment in an intensive care unit (code 8-98e). Among these, 2,542 and 1,124 cases were attributed to patients with malignant gliomas, accounting for 2.4% and 2.1% of the total, respectively. This implies that approximately 350 hospital cases per year were not considered in the present analysis. Moreover, we are also unable to determine whether specialized outpatient palliative care was initiated for malignant glioma patients. These data are not collected as part of routine. Outpatient palliative care, particularly in the context of chronic, progressive illnesses like malignant gliomas, plays as important role in patient management, symptom control, and quality of life enhancement. Its omission from the study potentially overlooks a significant component of the care continuum for these patients. (4) We used Pearson’s chi-squared test to evaluate whether the observed differences in proportions in our database can easily be attributed to chance alone. The Neyman-Pearson framework of statistical hypothesis testing rests on the long-run behavior of test statistics, i.e. it implies the notion that an experiment or scenario could be repeated many times. This notion is almost always unrealistic in medicine, however the tool statistical hypothesis test still proves useful to make reasonable decisions, in our case regarding associations of categorical variables. Thus, the Neyman-Pearson framework and statistical tests like Pearson’s chi-squared test offer a structured approach to drawing conclusions from data, even in fields like medicine. These tools provide a framework for making informed decisions based on the evidence available. Though many readers may be familiar with effect sizes we think that differences in proportions are even much easier to understand, and thus, we should like to keep them. (5) While interpreting the data, it’s essential to consider the probability of unaccounted confounding variables that are not evident in the InEK dataset. Treatment guidelines for individuals with malignant gliomas primarily rely on factors such as molecular genetics, the overall health condition assessed through the Karnofsky performance score, tumor size, location (especially concerning eloquent structures), and patient preferences, which unfortunately are not included in the available data [15]. Nonetheless, the InEK dataset provides insights into the contemporary landscape of neuro-oncological and palliative care between 2019 and 2022. (6) The data analyzed covers the period of the SARS-Cov2 pandemic in Germany. The number of cases and frequencies appear to be relatively stable for the most part over the period. Nevertheless, the pandemic certainly represents a potential confounder in treatment, but on the other hand, these potential effects can hardly be estimated based on the data. (7) The present data offer a broad overview of inpatient care in Germany. They do not specify the timing of patient integration into palliative care, making it challenging to discern whether the care provided was early integrated palliative care or end-of-life care. (8) As general palliative care is inherently within the scope of every doctor’s medical treatment responsibilities, there is no additional data related to this aspect that could be derived from billing data. (9) Any changes in healthcare policies, reimbursement practices, or guidelines during the study period that could affect the provision of care are not accounted for. Such changes could have direct implications for treatment availability, the approach to palliative care, and overall patient management strategies. (10) The reliability of the findings is contingent upon the accuracy and consistency of the coding practices used in the InEK database. Misclassification or inconsistent coding of diagnoses, procedures, or treatments could introduce biases or inaccuracies in the results. There is also the potential for variability in how different hospitals or healthcare providers interpret and apply coding guidelines, which can affect data uniformity and comparability. (10) In our analysis, we found that about 12% of deceased patients underwent tumor resection, 3% received chemotherapy or immunotherapy, and nearly 7% received radiation therapy during their last hospitalization. Although the time interval between antitumor therapy and death is a crucial quality indicator for oncological treatments, it cannot be determined using the InEK data [4].

## Conclusion

This comprehensive analysis of inpatient care for patients with malignant gliomas in Germany from 2019 to 2022 underscores a critical gap in the provision of specialized palliative care. Despite established guidelines advocating early palliative care integration, our findings reveal that only about 10% of hospitalized malignant glioma cases received such care, with a mere 40% of in-hospital deaths involving specialized palliative interventions. This discrepancy highlights a significant divergence between recommended practices and actual care delivery. The observed variations in care based on age and gender further emphasize the need for tailored approaches in palliative management. Our study calls for heightened awareness and action to bridge these gaps, ensuring that all patients with malignant gliomas receive the comprehensive care they require, reflective of their unique clinical and personal needs. Future research should focus on identifying and overcoming barriers to the implementation of early and effective palliative care, taking into account the diverse needs of this patient population.

## Data Availability

No datasets were generated or analysed during the current study.

## Declarations

of generative AI and AI-assisted technologies in the writing process.

AI-assisted technology was neither used for the generation, evaluation or interpretation of the data presented in the manuscript, nor for the creation of text, figures or tables. AI-based tools (chat GPT) may have been used to improve language and text readability.
